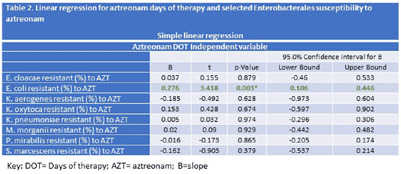# Impact of an intervention that decreased aztreonam DOT on Enterobacterales’ susceptibility to aztreonam

**DOI:** 10.1017/ash.2024.160

**Published:** 2024-09-16

**Authors:** Jose G Castro, Adriana Jimenez, Tony Anderson, Jennifer Quevedo, Bhavarth Shukla

**Affiliations:** University of Miami; University of Miami Health System

## Abstract

**Background:** Aztreonam (AZT) is frequently used for the treatment of Enterobacterales-related infections, particularly for patients with penicillin allergy. We aimed to analyze the impact over time of changes in AZT Days of therapy (DOTs) on AZT susceptibility from some Enterobacterales after a multifaceted intervention to improve antibiotic management at a University Hospital in Florida. **Methods:** The study took place at a 560-lbed academic hospital in Miami, FL. A multifaceted intervention was implemented in this hospital to improve antibiotic management of patients with reported allergies to penicillin. The intervention included use of algorithm-based guidance, education, and feedback to providers. The analysis period spans from 2018 (pre-intervention) through 2022 (post intervention); 2019 was considered the wash-out period (Figure 1). Quarterly data for AZT-DOT and percentage of resistance to AZT for Enterobacterales were collected as part of the normal operations of the antimicrobial stewardship program (ASP) using the infection control module integrated in the electronic medical record (Epic Bugsy). DOT and Enterobacterales antibiotic resistance to AZT was analyzed using linear regression in SPSS. **Results:** We identified a decrease in DOT AZT and percentage of AZT resistance from E. coli during the study period (Table 1). This intervention led to AZT DOT’s decrease from a quarterly average of 24 DOTs in 2018 levels to a sustained quarterly average of 4.3 DOTs for 2020 to Q2 2023 (decrease 80%) Antibiotic resistance to E. coli AZT changed from a 26.6% to 21.5% (19% decrease) (Table1). Simple linear regression identified a decrease in percentage of E. coli resistance to AZT associated with a decrease on AZT DOT (P-value 0.003), there was no association for other Enterobacterales. For each unit of decrease in AZT DOT there was 0.3% decrease in percentage of E. coli resistance to AZT (Table 2.) **Conclusions:** A decrease in AZT DOT during the observation period was associated with a decrease in E. coli resistance to AZT. Interventions aimed to decrease inappropriate antibiotic use are pivotal part of the fight against antimicrobial resistance; in our study we report a decrease in E. coli resistance to aztreonam related to decrease in the use of this antibiotic using education, guidance, and feedback to providers.

**Figure:**
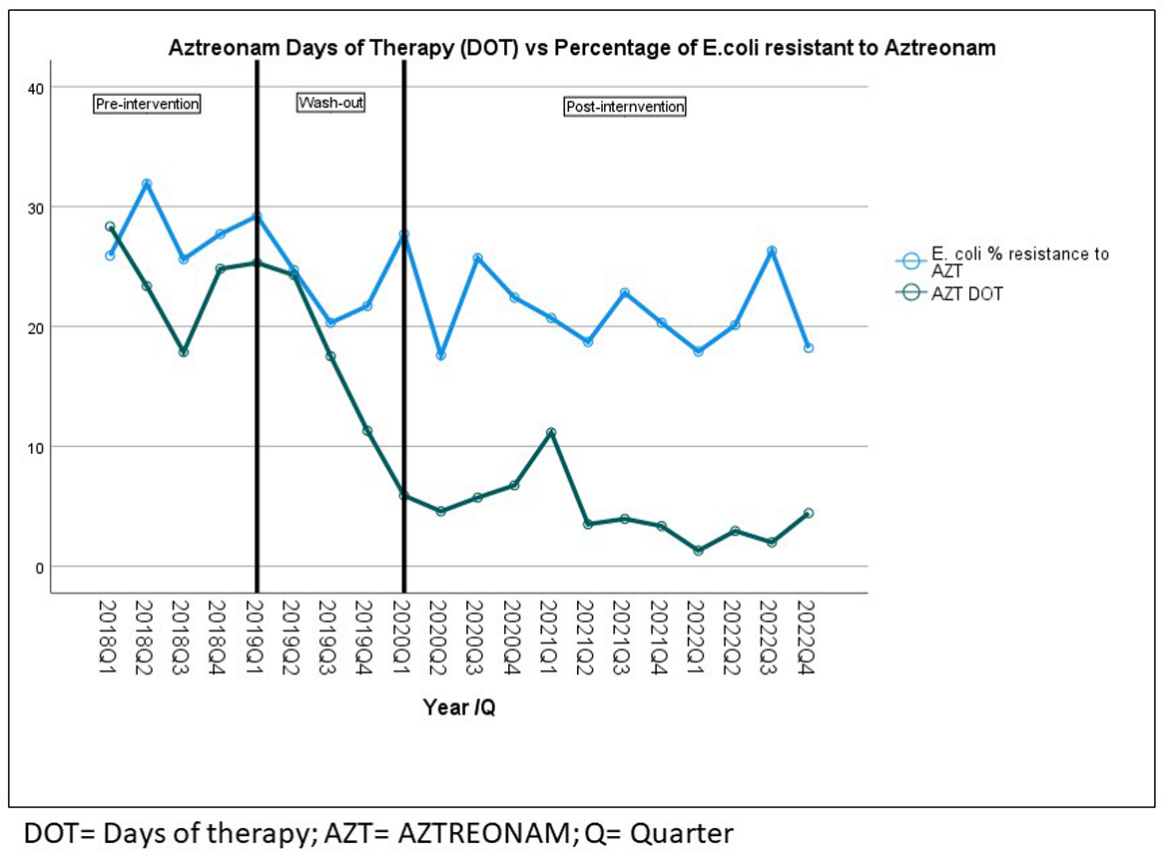

**Figure:**
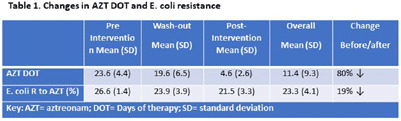

**Figure:**